# Upholding human rights in mega sports: A study of governance practices within the IOC and FIFA through the lens of the Ruggie Principle

**DOI:** 10.1016/j.heliyon.2024.e35607

**Published:** 2024-08-02

**Authors:** Shuang Lin, Asif khan, Yu Song

**Affiliations:** a School of Economics and Management, Shanghai University of Sport, Shanghai, China; b School of Law, Hainan University, Hainan, China

**Keywords:** FIFA, Governance practices, Human rights, International organizations, IOC, Mega sports, Ruggie principle, Sports governance

## Abstract

The human rights and mega sport events (SMEs) discourses gain massive importance and have grown immensely in recent years. Most of the time, the sports governance bodies are responsible for administration and management of these sport mega events. Keeping in consideration the situation regarding increasing involvement, the study focuses on the field of sport along with pleasure and basic rights. Through re-evaluation the commercial factors of sports, the mega governing bodies of sport (IOC & FIFA), and researchers discuss what is referred to commonly as Ruggie Principle, and both would be applied to IOC & FIFA practices, operation, and management regarding their events. This study is essential as it emphasizes the contribution and importance of carrying out human rights impact assessments, particularly in the context of hosting rights. The survey's findings reveal that human rights and due diligence assessments are integral components of organizations such as FIFA and the IOC. This study, being an empirical investigation, allowed us to arrive at some normative conclusions that had significant implications for practices and policy issues. Additionally, it laid the groundwork for future research in the crucial area of SMEs globally.

## 1 Introduction

**Fig. 1:**
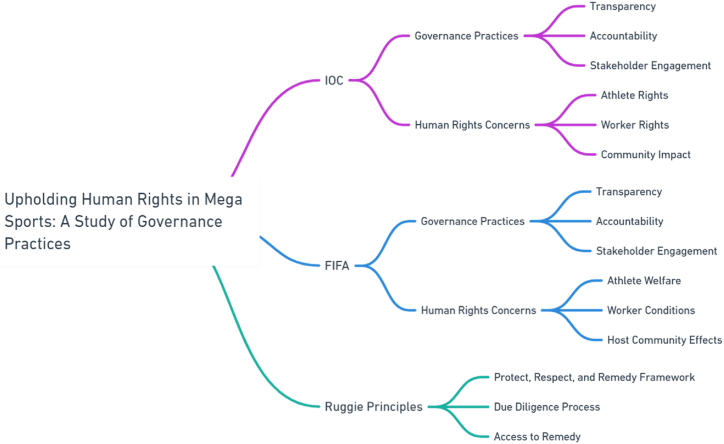
Summary of research.^1^.

**Fig. 2:**
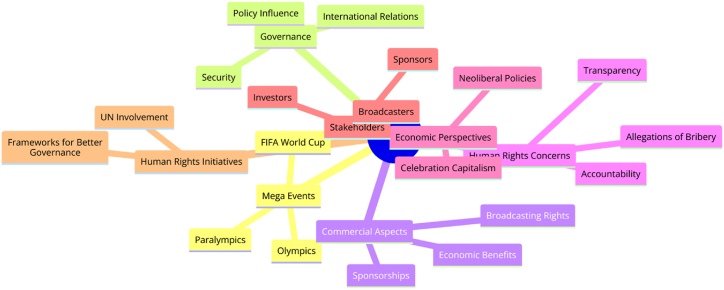
Review of literature.^2^.

**Fig. 3:**
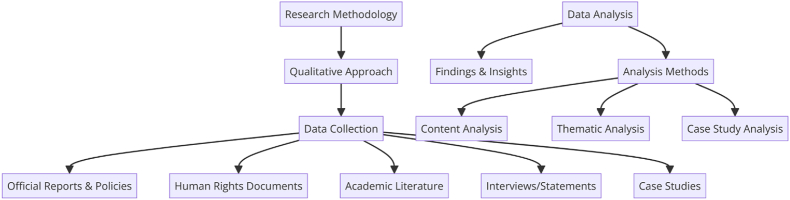
Research methodology.^3^.

**Fig. 4:**
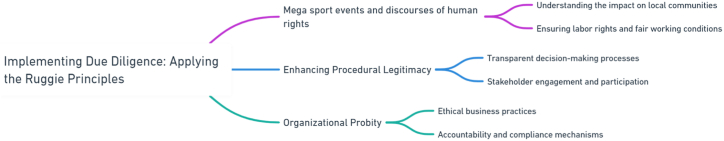
Implementing Due Diligence.^4^.

Management of mega sport events are strongly interlinked with diverse leisure, tourism, consumption, sport facilities and sport participation. The empirical evidence from existing and growing literature regarding mega-sport events and basic human rights, United Nations presents the respect, safety, standards and model like Ruggie Principle (RP). In this study, researchers tried to examine the practical implementation of said framework in the context of FIFA & IOC mega-events. The human rights impacts assessment can observe Debeco (2009) [1] the understanding the HRI of upcoming mega events with better policies, implementation and ensuring healthy participation of numerous actors. The interactional aspects of human rights and sport generate various challenging and profound claims. Indeed, starting comparison among legal and conceptual paradigms, mega sport events & human rights appear in common. Well established claims regarding complicity and supporting these charges by the governing bodies of sport in host nation's human rights abuse. A lot of factors include such as forced labor during managing sport events, displacement during preparation, building larger infrastructure, culture if impunity with sporting bodies appearance before, during and after sport mega events to specific countries [2,3] and apparent in ability of international and national sport governance bodies effectively socio-cultural, racism and homophobia [4]. (see Fig. 1, Fig. 2, Fig. 3, Fig. 4).

Subsequently, various researchers have participated regarding the nexus among sport mega events and human rights. This research study focuses on Mc Gillivray and colleagues (2019) [5] policy orientation agenda. This evidence-based study tried to cover the gap regarding practical implementation of human rights impacts assessment with regard to governing bodies of mega sport events. In this study, researchers bring to light insights and conceptual understanding from sociological perspectives, human rights activities, leisure and event studies, official understanding reports and socio-legal aspects of research. To coherent discussion and comprehensive discussion two research question were poses this study such as.

1. Implementation of guiding principles of human rights during the mega sport events of IOC & FIFA
2. Basis of human rights implementations and mainstreaming of human rights became the operational priority for mega sport governing bodies.

With respect to Ruggie Principles this study supports that due to human rights and diligence assessment becomes an organizational mainstay of event organizers such as FIFA & IOC operations. Borrowing understanding from various national and international reports regarding human rights, this study provides tentative ways for how these would be implemented to ensure better sport bodies apply on the hosting nations to act according to their position and practices human rights. The study contends the governing bodies and nations should underpin and act according to systematic process and ensure human rights mainstreaming throughout the events.

### 1.1 Why focus on this research

The research focuses on the governance practices of the International Olympic Committee (IOC) and the Fédération International de Football Association (FIFA) in relation to human rights within the context of mega sports events (SMEs), guided by the Ruggie Principle. This focus stems from the increasing importance of discussions around human rights and SMEs, as these events often involve complex interactions between sports governance bodies, host nations, and various stakeholders, leading to potential human rights challenges. Issues such as forced labor, displacement, socio-cultural racism, and homophobia during event preparations highlight the need for a comprehensive understanding of how these governing bodies operate and manage their events with respect to human rights. The research aims to address gaps in practical implementation of human rights impact assessments by these organizations and to provide insights into the operationalization of human rights within the sports sector. By examining the adherence to the Ruggie Principles by the IOC and FIFA, the study seeks to contribute to the development of policies and practices that ensure the protection and promotion of human rights in the context of SMEs.

## 2 Review of literature

The researcher concerned private organizations such as IOC & FIFA, because ultimately said sport bodies are primarily responsible for manning and overseeing the mega events such as Paralympics, Olympic Games and FIFA world Cup. Thus, the prime focus of this study is on the most influential and largest non-governmental organization.

The head offices (Switzerland) of FIFA & IOC as private of organizations for planning, maintaining, and organizing mega sport events worldwide. The bereft of legal position according approved procedure, they have the legal position that exerts huge impacts on regional development. Organizations like IOC & FIFA have gained unprecedented importance and have significant influence on policy making, international relationships, trades and commercial involvement along with security aspects among others. FIFA continues growing in all aspects and is considered the largest non-governmental organization in known human history [6]. The involvement of numerous aspects of private organization, these sport bodies characterized more as commercial in nature. Intensive commercial involvement, economic profits and benefits add more and more growth in assets and wealth, but these organizations cannot share with members or shareholders like other organizations.

Commentators, journalist and academic scholars often pointed this out from time to time but these organizations whilst diverse related to lack of transparency, accountability, allegation regarding bribery that significantly associated with human rights concerns. In the context of this study and Horns (2018) [7] views regarding human rights awareness are critical and most appealing aspects of mega sport events and have received great attention in recent decades globally.

The economic aspect and commercial nature of FIFA & IOC would be understood in the light of neoliberal policies which molded international sport over the years. This converts elite sport among lucrative industries for sponsors, investors and broadcasters accordingly. International sports events such as the world cups, represent various brands globally, and governing sport bodies like IOC & FIFA become the key generators of capital for investors, brands, stakeholders and broadcasters. For example; the rights of broadcastings for FIFA 2010 mega sport event conducted in South Africa revealed that the 60 % of revenue of all FIFA income which is about to 3890 million [8].

IOC & FIFA have become the leading mega sport organizing organization in the world but also have similarities with the economic perspective. Researcher highlights that IOCignored aspects regarding sponsorship, broadcasting, partnerships, and maximization of profits which theorizes as *“celebration capitalism”.* The sponsors of Olympic Games like world cups pay huge amounts of fees for their rights of broadcasting, branding, and ads among others. In this regard IOC adapts the commercial and capital orientated [7].

Mega sport governing bodies are significantly indulging in the business of commercial marketing on an international level and making huge profits through various aspects of the mega events [8]. The important question is that during the process of making profit through sponsorships, advertisement, broadcasting and other factors of mega sport events, human rights issues are dealt with by these governing bodies and host nation countries or ignored according to their economic benefits.

For example, some critical reviews point out that these mega sport governing bodies in fact shield away from realization and accept responsibility of human rights activities during mega events and believe that is the responsibilities strongly associated with host countries. Number of existing literature and empirical academic reports regarding human rights observation during mega sport events highlighted the incredible growth in mega events. The line with the arguments by McGillivray et al. (2019) [5] that various members and stakeholders supports the efforts made by the United Nation regarding human rights. There are also comprehensive frameworks associated with better organizing, governance, formalization, and participation agendas with respect to human rights activities not only during mega sport events but also in the urban development, mobilization, and transformation during the process of SMEs.

### 2.1 The theoretical framework

The theoretical framework of the research is centered around the Ruggie Principles, which are comprehensive guidelines developed by the United Nations for addressing human rights within the context of business and commercial activities. These principles outline the responsibilities of corporations (including non-governmental organizations like IOC & FIFA) to respect human rights, the role of states in regulating and enforcing human rights protections, and the need for effective remedies for human rights abuses. The research incorporates sociological perspectives, leisure and event studies, and socio-legal aspects to examine the practical implementation of the Ruggie Principles in the management of mega sports events by the IOC and FIFA. It also involves an exploration of due diligence and human rights impact assessments (HRIAs) as organizational practices for these sport's governing bodies. The theoretical framework also extends to the concept of *“mainstreaming”* human rights [9] within these organizations, promoting a systematic integration of human rights considerations into all aspects of their operations. This includes understanding the regulatory role of various international treaties and agreements on human rights and how they apply to both public and private entities involved in mega sports events. Overall, the theoretical foundation of the study is multidisciplinary, incorporating elements of international law, business ethics, sports management, and human rights advocacy to address the complex interactions between mega sports events and human rights issues.

Furthermore, in order to validate the hypotheses and sub-hypotheses, the research analyzes case studies of such recent mega-sports events as the FIFA World Cup and the Olympic Games. The analysis includes data regarding human rights complaints filed, resolutions achieved, and the effectiveness of the due diligence process at mega sports events. The research will use quantitative data on the number of reported human rights violations, the nature of these violations, and the procedures taken by the event's organizers to respond to these allegations. Primary and secondary sources, including representatives of human rights organizations, sports' governing bodies, and the involved communities, will collect the data. The data-driven research will enable an understanding of both the best and worst practices in the real-life implementation of the Ruggie Principles and the protection of human rights at mega-sports events.

## 3 Research methodology

The research methodology for this study on the governance practices of the IOC and FIFA in relation to human rights, specifically through the lens of the Ruggie Principle, is not explicitly detailed in the provided manuscript sections. However, based on the nature of the study and the topics covered, it can be inferred that the methodology likely involves a qualitative approach. Qualitative research in this context would involve analyzing various sources such as official reports, policies, and guidelines from the IOC and FIFA, human rights documents, academic literature, and possibly interviews or statements from stakeholders involved in mega sports events. This approach would allow for an in-depth understanding of how the Ruggie Principles are applied (or not) by these organizations and the impact of their practices on human rights. The research might also include case studies of specific mega sports events to examine instances of human rights issues and how they were addressed by the governing bodies. Analysis of legal documents, press releases, and public statements could provide insights into the organizations' commitment to human rights principles and the effectiveness of their implementation strategies. While the exact qualitative methods (e.g., content analysis, thematic analysis, case study analysis) are not specified, the study's aim to explore the practical implementation of human rights impact assessments and the operationalization of the Ruggie Principles suggests a comprehensive examination of qualitative data from multiple sources.

## 4 Re-examining the Ruggie Principles (RP)

United Nations Human Right Council (2011) [10] promulgates a comprehensive principle which provides clear guidelines with respect to human right and commercial implementing are commonly known as Ruggie Principles. These principles represent at national and international level, and serious effort to provide guiding principles, to regulate legal capacity and boundaries among private sectors, organization, and events regarding human rights regulations. Former representative of united nation secretary general, John Ruggie articulate these principles and try to regulate human rights in the non-governmental organization during their involvement of business, developing enterprises and amplified operationalization. Ruggie presented the policy and conceptual framework to business and organizing bodies regarding human rights and highlighted duties and responsibilities according to the nature of situation, responsibilities arise. Ruggie principles consider responsibilities to ensure protection regarding abuses by business, and parties’ responsibilities with more affective mechanism of remedies. In the result significant outcomes observed at national and international level regarding gaining peace, harmony, productive bindings on treaty and business regarding human rights. The Ruggie principles one among the most important *go-to* guide with the perspective of human rights in the discourses of business and mega events [11].

Imprimatur of the global community such as organization for economic cooperation and development (OECD) [12] and united nation human rights council (UNHRC) [13] governing bodies established legal structure and policies regarding human rights in private organization are evolving together. There are interlinked aspects of Ruggie Principle such as i. Corporate responsible for respect to basic human rights, ii. State responsibility regarding human rights, iii State is responsible and has obligation to provide remedy. Noteworthy characteristics of principles, framework regarding ensuring human rights in the events of non-governmental, private sectors and business at various locals. Researchers significantly argue that the interlinked connections of private regulations and state laws. Comprehensive coverage, broader scope, implement these principles in all events, business, in transnational events regardless of their existing situation and ownerships [14]. Due to universality and adjusting nature IOC & FIFA also fell squarely and fulfilled the parameters defined by RP.

### 4.1 Ensuring human rights: the role of states

Ruggie Principles is considered as the first thematic draft which provides fundamental legal structure for international governing bodies and non-governmental association with regard to human rights during the process and sets primary responsibilities and liabilities to the states to ensure them. The classical vertical axis that deals with state and individual basic laws, ascribes the responsibilities to protect, respect and fulfill all conditions required for human rights. These fundamental situations are well articulated with lexical, legal, and regional human rights principles. Private sector's, political, social and traditional affairs has resulted in a paradigmatic improvement with reoriented relationships among these sectors [15]. While the nations or states assumed the obligation continually as duty bearer to provide human rights commitments, the improvement of recognition in the private sphere significantly impacts and shapes the role with wider problems of economic governance, legal regulation and responsibilities.

On the other side these principles endorsed that while state was not responsible regarding human rights abuse by non-governmental sector (UN, Principle 1), but they would nonetheless ignore legal responsibilities, where these abuses can attached to them or if they are unable to perform appropriate initiatives to investigate, prevent and redress private bodies abuses. Italy has failed to ensure basic measures regarding environmental pollution of chemical industries and interfering with the applicant's private and family life. In the same way in *Fadeyev Vs Russia* [16] mater the court decided that the responsibility of state arises with the filer of regulation of the private sector. The said case directly emanated regarding environmental problems, but nonetheless revealed that the necessary actions regarding misconduct of private sectors are not ignored from regulatory authorities of states [17]. The action regarding commercial bodies and private organizations has also the equal implication of both concern governing bodies such as FIAFA & IOC and they are bound to observe human rights aspects in the wider success of mega events.

In recent decades the momentum regarding human rights at international level has proliferated and various agreements, treaties and commitments are outlined accordingly. These agreements and treaties enhance the regulatory role of public as well as private organization, business and governing bodies. For example, the United Nations committee on economic, social and cultural rights, United Nation Rights of the child [18] oversees the whole process in states and during special circumstances. These organizations not only ensure the targets but also regulate the action regarding the issue occurring within their jurisdiction. The basics of the guideline for both governing bodies has reassertion that the private organization falls outside the legal axis from the obligation imposed human right declaration provided to individuals and states. The former governance body supports that state should play its role and prevent private sector to avoid children rights and observe measure of regulation, policy which frame how different private sector can influence on the rights of children. Similarly, other governing body administrations accept legislation, and other suitable measures to maintain safety regarding violation [19]. Therefore, non-governmental sectors would no longer be covered because of non-state character, and the state should not be blind eye towards private activities of this sector.

The Ruggie Principles also cover such types of sentiments, and clearly contend that all states must act as per policies and established laws and ensure human rights observation by private sectors and organizing bodies within their jurisdictions (Principle 3). Furthermore, significant action must be taken to ensure that all the private sector, their owners, sponsors, supporters are controlled and monitored by states with regard to human rights observation (Principle 4) [20]. State prime responsibilities are to ensure policies regarding private and business communities not only to protect their actions but also guide them to maintain structural procedure. Therefore, reaffirm the establishment of legal orthodoxy by the Ruggie Principle assuming the basic responsibility of state. Legal application, protection of human rights, unclear activities of governing bodies such as FIFA & IOC can start responsibility, if these actions are attributed to the state or exacerbate the impact of violating action [21].

#### 4.1.1 Enforcing compliance: FIFA and IOC strategies for host states

The Ruggie Principles have provided a basic groundwork for international managerial entities like FIFA and the IOC to tackle human rights issues within mega-sports events. These principles define the duties of states to protect, respect, and fulfill human rights within their domains. State duties with respect to human rights in the area of business activity are to protect against human rights abuses by third parties, including business enterprises, through appropriate policies, regulations, and adjudication developed in the relevant jurisdiction [22]. Therefore, it is imperative for state parties to establish and enforce policies and legal frameworks that guarantee the respect of human rights by all parties involved. All transnational corporations making appearances at any stage of the mega sports event must fulfill their duties to their states and respect the human rights of all within their jurisdiction. These include the sporting event management bodies, FIFA, and the IOC [23]. To ensure compliance with these principles, FIFA and the IOC can incorporate human rights standards into their bidding processes for mega-sport events. The two international institutions can demand that a country willing to host a mega-sport event achieve some level of human rights standards and provide information on what it has done to meet such standards. This strategy implies that those countries that would be pursuing opportunities to host mega-sport events must ensure that they have good relations between government institutions and society and must have developed plans to address all human rights concerns. Thus, FIFA and the IOC would only allow countries with efforts to produce human rights to host the mega-sport events [24].

With respect to a uniform normative structure, states are generally not bound to any within the realm of human rights. However, contractual agreements between the IOC, FIFA, and the host city or state can further secure compliance after choosing the host. The nature of the agreement allows for establishing binding clauses related to human rights, particularly those prohibited under international law and its principles relating to labor conditions, non-discrimination, and prevention of abuses [25]. In the event of a violation of a clause, the agreement may stipulate a predetermined course of action, which may include financial and other sanctions on the part of the parties, and in severe cases, the revocation of the hosting permit. Regardless of the contractual agreement's specific content, the possibility of negative outcomes makes it an effective tool for compelling the host state to address human rights in the preparation and execution of mega-sports events [26]. Monitoring mechanisms are one of the most effective tools for ensuring ongoing compliance. Therefore, FIFA and the IOC should establish regular audits as well as assessments conducted throughout the event's entire life cycle. For these purposes, representatives of civil society organizations and human rights experts should participate in independent oversight bodies. They would provide transparency for the aforementioned processes and ensure accountability for the assessment activities. These bodies would ensure that a host state effectively implements the policy and reacts on time in case of any human rights challenges that may arise [21].

Moreover, host states can benefit from capacity-building measures and technical assistance in developing and implementing adequate human rights policies and practices. Training local organizers, government officials, and stakeholders will ensure they possess sufficient knowledge of human rights measures and are well equipped to adequately incorporate them into the event's planning and management. Stakeholder engagement is another vital aspect of enforcement. FIFA and the IOC should mediate a broad dialogue between host states, local communities, civil society organizations, and other stakeholders. This will guarantee the inclusion and consideration of all perspectives, thereby establishing a foundation for informed decision-making concerning mega-sports events [27]. Another important step is to design and establish accessible grievance mechanisms for the individuals and communities that suffered from the event. By implementing these strategies, FIFA and the IOC adhere to the principles outlined in Ruggie's framework. Moreover, by developing such an approach, these organizations contribute to advancing and protecting human rights within the context of mega-sports events in the host communities. These practices will not only prevent the development of human rights abuses associated with mega sports events, but also ensure that the local population remains unaffected after the event concludes [28].

### 4.2 Protecting human rights: the role of corporations

This thematic aspect of Ruggie Principles explicitly focuses on the private sector which is the most relevant with the context of IOC & FIFA. The aggregate of 14 sub-sections or principles intensively talk to deal with non-governmental organization. Furthermore, these principles expound numerous responsibilities which include business needs with respect to human rights (Principle 11), avoiding adverse violation of human rights through planning and action (Principle 13), diligence the process to identify, mitigate and prevent regarding adverse consequences (Principles 15 & 17), embed adherence within the policy agreements (Principle 16) and impact assessment pertaining to private sector activities in order to understand the risk attach with their actions [29].

The aspect of Ruggie Principles has never evaded scrutiny. The nuances of terminology in this assessment enunciated the perspective and *responsibility* that give them importance. Wettstein (2015) [30] points out those practical results are the basics rights at international level that strongly associated with the role. The findings of anathema deal with the legal aspects law observing responsibilities and same way revolves and creates ambiguity. Both aspects operationalized within objectivity discretionary and malleable parameters but their deficiency. The practical implementation of respective *responsibilities* and *duties* neither are nor fixed nor identifiable.

This situation is confusing and other approaches such as robust rebuke by the Ruggie Principle determine the responsibilities of the private sector to ensure human rights. Bilchitz (2013) [31] also highlighted that cogently fails to account for the due legal responsibilities and give effect to third parties such as commercial corporate actors.

*The states are required to maintain order and enforce legal obligations that were established expressly through various national and international treaties accordingly. The logic behind this act is the state is responsible to protect individual’s human rights, and observe international laws which necessarily entail by the nations that private organizations such as corporations and have legal bindings with respect to human rights.* [31].

However, the well-established and practical concerns of various researchers and scholars regarding the need, importance, outcome and discourses of Ruggie Principles, the most important recommendation that the private sector observes due to diligence impact assessment and compatibility with activities of human rights principles (Principles 17–21). Ruggie Principles are important yet need more dissemination and implementations accordingly [32]. Researchers argue that:

*Risk and costs regarding appropriate due diligence endeavor along with the importance of available evaluation procedure cannot provide sufficient evidence that brings on conclusions regarding effectiveness of corporations trying due diligence, the ethical targets of the private sector or become a decisive aspect for the importance of which corporation would be attributed to human rights due diligence process.* [33].

However, FIFA & IOC with the context of prime activities such as transportation and adoption of robust due diligence and assessment of impacts may affectively with existing process of deciding things in the organizations. Many of the FIFA & IOC generate serious questions with respect to commitments. In easy reduction of terms, the impact assessment involving HRIAs revealed an explanatory foundation that always helps in decision making. The ultimate success involves numerous aspects such as time of decision, preparing the report and making assessment itself and also robustness and involvement of different stakeholders. De Beco, 2009 [1] discusses how HRIAs yield various benefits such as enhanced compliance of human rights standards, integration of policy and implementation of human rights, appropriate decision making, effective accountability, participation, empowerment and bring human rights at the entire focus of policy development process. This process through the understanding of risks, costs, benefits, disadvantages and advantages significantly attached with decision, careful examination, availability of evidence depended upon the process of acceptance and rejection of decision. In the same way, De Schutter, 2011 [34] points out that guiding principle on HRIAs regarding investment and trade sets various significant elements which cover these assessments. The necessity for methodological designs, that makes explicit involvement for normative content, obligation which involve within models.

This research contented requirement for assessment of impacts among the various aspects of Ruggie Principles that have much potential for better implication of human rights activities of IOC & FIFA. Furthermore, as discussed, the ultimate success of such kinds of initiatives is significantly associated with the culture and adoptive nature of organizations and governing bodies.

### 4.3 Ensuring access to remedies

The next important aspect of the Ruggie Principle is associated with access to remedial measures. The states always take appropriate measures to provide facilities and ensure availability of necessities through various means such as administrative process, judicial involvements, legislative actions that provide assistance to states within their jurisdiction regarding human rights assurance. Allowing for remedial measures is fundamental recognition that states bear basic responsibilities and guarantee the rights. Access to remedial measures and the private sector establish effective grievance mechanisms for communities and individuals. According to this declaration states must arrange sufficient measures to reduce the aggrieved groups, communities and provide private sector.

Within the PR reinforcement non-judicial mechanisms raise the ADR and the whole process involved with. Various aspects as accessibility, legitimacy, and equity among others refer to Ruggie Principal aspects (Principle 31). Although the various human rights agreements, treaties failed to achieve important indicators and correlated obligations. Recently McGregor and colleagues, 2019 [35] argued that improving reliance on ADR from this perspective. As highlighted, that:

*The unequal power distribution among parties particularly involves human rights problems and is strongly associated with these concerns. In the due process and concern the most influencing party such as private organization or state may be invested in the due process. The deficiency of equal* opportunities *for all parties imbalanced the situation and created complications. To settle the relevant concerns both the parties depended on court procedures regardless the importance, sensitivity and strength of cases.* [35].

Furthermore, the above statement illustrates a common problem with relations of unequal power within the context of courts of law and human rights concerns. Disparities in power, such as those held by large private organizations, public authorities, or nation-states versus significantly less powerful individuals or small communities, heavily influence the processes by which human rights concerns may be duly resolved within the legal system. Typically, whichever side holds more power is able to ultimately manipulate due process, decision-making, and any other legal niceties in their favor, thereby compromising fair and equal opportunity for all [36].This naturally leads to a deadlock in the process of resolving human rights concerns, which means that both parties will attempt to resolve the conflict or dispute through the courts of law. The nature of the courts of law and the established legal framework allows for such matters to be resolved, whether they are relatively straightforward, as in most civil disputes, or extremely sensitive, as in matters of human rights [37].

## 5 Implementing the Ruggie Principles: due diligence and impact assessment

Considerable the basic characteristic of RP present three specific reasons regarding the due diligence practices and HRIAs can be the organizational mainstay within both governing bodies of mega sport events such as FIFA & IOC including other sport bodies in broader perspectives.

### 5.1 Mega sport events: examining human rights discourses

In recent years, the proliferation of human rights in mega sport events is the interface and engagement of the events. The phenomenal influence of private governing bodies in mega sport events and strongly argued that they feel they are not accountable for their actions, management, and policies like other aspects of social justice. Indeed, Amis (2017) [38] brings into account that with the perspective of mega sport events which are associated with arranging sport events by IOC & FIFA that:

*During the period of the last two decades unprecedented challenges and patterns regarding human rights emerged. The issues are multidimensional and cover various aspects such as resettlement and housing rights in the process of land acquisition, labor rights, development with adjustment, internal & external migration, exploitation during preparation of infrastructure & constructions, the process of arranging food, goods, & products, and above all in the process of services timing, facilities and arrangement of safety among others.* [38].

Perhaps, the severe consequences regarding violation of human rights during the due process of arrangement of mega sport events is significantly reported during the mega word cup preparation in Qatar 2022 [3]. The large scales deaths of workers in the process of mega events clearly indicates that the workers often face wider economic, wage abuse of health, physical and emotional exploitation. In parallel, various pieces of evidence bring into notice the numerous tangible actions of governing bodies regarding such incidents accordingly. During Beijing Olympics in winter 2022 highlighted aspects regarding the management of this mega sport event such as elimination expression, curtailment, and rights. On an average scale different actions are taken by IOC on the violation of human rights and try to ensure international treaties and manage the issues accordingly. Of course, it was not suggested that the international sport governing bodies hold the legal accountability of states in this regard. But they may provide their services and fulfilled responsibilities by reminding states of their obligation and duties to ensure better arrangement along with human rights observations accordingly. Keeping in consideration the situation, This research strongly argued that combination within the whole process of organizing events offer more grounded by human rights declarations [39].

Given that IOC & FIFA delivered standard of due diligence and HRIAs must observe such actions. Recently, in the 2021 iteration, the charter of Olympic proclaims that the event is considered social responsibility and respects all ethical considerations globally [40]. The respectable with better observing and arranging spirit of a sport man during due process and enjoying freedom without discrimination, such as on the basis of sex, color, religion, language, political affiliations, origin among many others status (Principle 6). Furthermore, FIFA committed to better protection of human rights activities during their events and reduce discrimination at advance level with all respect. John Ruggie (2015) [2] also examines the manner of FIFA governing bodies and presents how they manage human rights during their event at regional and international level. Ruggie among his 25 recommendations clearly mentions that FIFA and other mega sport bodies must observe the human rights activities at their best level and enhance observation during their operations and ensure appropriate remedy. This was reflected during 2021 FIFA status where FIFA ensured commitment with entire international recommended human rights suggestions [41]. In the same way IOC also showed commitments regarding observation of human rights during its events [42] and followed Ruggie Principle accordingly. The advance recommendations specifically articulated IOC responsibilities, respect to principles, identifying issues and ensuring human rights due diligence process.

With increasing awareness and observation both the organizations such as IOC & FIFA embed better arrangements regarding human rights activities within their organization during the managing of mega sport events. They galvanized strategic as well as operational visions, and deeply concerning how to improve and ensure human right action that make these events unobjectionable in all aspects. That's why this study discussed operationalizing due diligence along with HRIA requirements regarding Ruggie Principle, and making best efforts how these operational aspects transform into practical actions and become a durable reality of all mega sport events at regional as well as international level.

### 5.2 Enhancing procedural legitimacy

On the basis of comprehensive discussion, it is argued that Ruggie Principles adherence and the particular process in which both IOC and FIFA private mega sport governing bodies involved in the due diligence process of human rights activities during their events can improve its procedural legitimacy in the process of policies, implementation and decision making. In the past various legal as well as ethical observations and allegations were pointed out time by time regarding both organizations which make their activities and management questionable with respect. During the process of events such as Qatar world cup, FIFA & IOC award the hosting rights to an organization which has faced serious allegations regarding the violation of human rights. This includes *inter alia* the mega event of Beijing summer Olympic 2008, Winter Olympic 2022 and Qatar world cup 2022. A lot of questions regarding other mega events such as Sochi, Russia 2014 winter Olympic that collapse [43].

The sport mega events are continually attached with liberal as well as corrupt activities. Similarly, Jennings this is due to ultimate liberty in decision making of FIFA & IOC governing bodies that goes toward corruption and unfair debates. When the research looks at the Olympic history, the 2002 Olympic events bid scandal is the most powerful example of all this discussion. Reports revealed that a large number of IOC employees were involved with bribes or gift scandals, wealth from Salt Lake City and university scholarships among many others. In the results of the IOC scandal it was established comprising four IOC members and five members from outside the organization [43] for investigation and to avoid further misconduct in mega events. In the case of Rio de Janeiro Olympics 2016 serious punishments were imposed on the Olympic committee [44].

However, the observation of Ruggie Principles and its recommendations for both private organization such as IOC & FIFA engage more realistic, transparent, and following due diligence process in the planning, implementation and outcome of human rights in mega sport events. Before the introduction of Ruggie Principles, Ruggie, himself argued that due diligence is just an attempt to deal the risks regarding human rights, positional and actual activities of organizations and states, systematic cycle of role and responsibilities without priority or obligation at any level. In this perceptive United Nation declares that the rights and obligation determination during mega sport event is one among the most influential contribution is considers guiding principles [45]. When research talk about the commitment of FIFA regarding insurance of human rights, the organization is in always ongoing process and trying to observe due diligence that deal with risks, integration, decision making, actions and mitigate the harmful aspects and enhance effectiveness of all taken measures. Similarly, IOC observes and shows commitments in this regard. Al Hussein and Davis (2020, p. 15) [42] significantly provide evidences that the observation of due diligence by IOC categorically enhance the abilities and capabilities of IOC to deal with wider issue regarding human rights. By regularizing the procedures and commitments private organizations must monitor, mitigate issues and enhance productivity within regional and international human rights standards.

The natural question came into consideration that how and what due diligence constitutes and how various mega sport organizing bodies should adopt and carryout in their events. There is not any single framework or definite model that exists for policy makers, organizers and governing bodies underpinning the assessment procedure for human rights or due diligence of whole organizing mega events as unifying features. From the commercial or business point of view of both organizations such as IOC & FIFA this embraces an effective attempt to regulate all economic activities and approaches which regulates the whole process of mega events, areas, accurate evidence, and related aspects of stakeholders with cost and benefits activities assessed. This also considers important necessities that have human rights implications, proposed aspects, and measures with ultimate decisions. The OICD declares that in line with guidance due diligence traditionally plays its role to prevent the undue actions and planning that cause adverse impacts on individuals, organizations, and society. It also seeks the negative impacts on direct and indirect operations services, products and partnerships of various governing bodies [46]. The OICD also highlighted that due diligence must be an integral part of any private organization during the process of their decision making, managing events, involvement with various stakeholders of events and organizations [46].

Theoretical understanding and underpinning of human rights and its due diligence have been established comprehensively, but its practical operationalization and implementation is an important aspect that needs more attention and actions accordingly. A lot of questions in this regard occur such as who retains responsibility, what are the important considerations, procedure to adopt these considerations, time of execution, monitoring mechanism and standards of successful happening among many others. Therefore, the embedding targets of such recommendations and guiding principles for private sector including the bodies of mega sport event remains active for efficient implementation and further ensure human rights in their events.

The various guidelines and models of human rights bring into consideration and enhance awareness of what could be done and included during the assessment. For example, the Danish institute of human rights (2020) [47] provides 5 steps that would be underpinned during the process of human right assessment. These phases include as i) planning & scoping phase, ii) data collection and baseline development, iii) the assessment of impacts of projects and analysis of severity, iv) impact mitigation and management, and v) reporting and evolution of impact assessments.

Similarly, some other important guidelines are provided by Harrison and Stephenson, 2010 [48] and introduce eight phases for this which covers as fundamental requirements, scoping, screening, collecting evidence, analysis, and consultation, conclusion, monitoring and publishing. The presented five important features which directly unify the whole practice, the consideration of framework and laws for conduct assessment of positional and actual impacts of proposed decisions and recommendations. In the same way, participatory processes enhance accountability, provision of remedies, actual implementation of design mechanisms and proposed work plans for any event.

HRIAs and due diligence require some further operationalization including deep understanding of regional and international law regarding political, civil, economic, cultural, social and their procedural processes associated with human rights processes of mega sport events. After the integration of all aspects of human rights law the legitimacy of decision, actions, recommendations and procedure of adaptation for all private sectors especially governing bodies of mega sport events is more important with intensive positive outcomes and dealing with issues [49].

Furthermore, the acceptance and respect to human rights law and due diligence by FIFA & IOC pertaining not only commercial practices but also covers institutions equipped to understand and estimate human right impact assessment within their organization and also during the mega events they organized. During this process the manifestation of adherence alters the existing culture of institutional practices which requires and needs the time for commencement of human right to attain probity that ultimately Ruggie envisages through his Guiding Principle.

### 5.3 Organizational probity

For the endurance of governance bodies like IOC & FIFA organizationally equipped and ensure probity in due diligence and during the assessment of impacts, it is strongly suggested that governance bodies must observe and engage in mainstreaming process of human rights. Certain concrete, systematic and conscious integrate into the policy process of programs, plans, process, priorities and results of activities and actions of governance bodies. Concept regarding mainstreaming has been accepted universally and has gathered regional and international prominence since 1997 by the United nation secretary general Kofi Annan [50]. Since 1997 the perspective of human rights become in the mainstreaming and one among the most powerful tool within private sectors as well as intergovernmental organization for maintaining, ensuring and sustaining human rights aspects in their structure [51].

In other words, the International Olympic Committee and FIFA, as governance bodies, must fully integrate human rights considerations into their organizational framework, particularly in their due diligence and impact assessment processes. This is necessary to ensure integrity and respect for the norms of fair play in all its activities. For these bodies, adopting a mainstreaming approach to human rights would provide a formal framework, namely the systematic and conscious integration of human rights principles and standards into policies, programs, planning processes, priorities, and operational outcomes.

In 1997, the world began to widely use and recognize the concept of mainstreaming as a means of defining human rights. The current concept explaining mainstreaming is that it is a strategy for integrating the analysis capacities, vision, norms, and goals of human rights into the practices and policies of a community. This basic definition allows us to view mainstreaming as a tool or strategy that integrates human rights considerations into normal activities or multicultural operations. Alternatively, we can recognize mainstreaming as an approach, policy, or strategy that aids the private sector and intergovernmental organizations, such as the IOC and FIFA, in transforming human rights into a regular, normal, and fundamental aspect of their operations, rather than a peripheral, exceptional, or optional one.

The IOC and FIFA should mainstream human rights in order to prevent potential human rights risks associated with mega-sports events. Otherwise, these organizations could be held accountable for human rights violations, as they are responsible for ensuring fair labor practices, preventing discrimination, maintaining environmental safety, and respecting the rights of local communities. On the other hand, integration can promote the credibility and legitimacy of these organizations, as well as emphasize the maintenance of international human rights standards and obligations.

The mainstreaming of the process of human rights generates various operational and practical issues. Yeshanew 2014 [52], discussed the implementation of a number of policies or some of the policy branches or organization sub sections that have certain issues during this process. This specific pertinent for IOC & FIFA who deal with this multilayered level and the Ruggie Principles are silent with the regard of mainstreaming. It is due diligence and contended that during the implementation of due process both governing bodies would cover mainstreaming that have few gaps in explanation and practical action. Oberleitner, 2008 [53] points out that mainstreaming of human rights is effectively observed and various practical organizational events must be occurring. This also covers the provision of training, staff, infrastructure and administration within the organizational procedure of human rights considerations.

Although, Ruggie Principles does not highlight the concept of mainstreaming and due diligence that creating impact assessment, the fundamental coverage of human rights observed by all organizations especially both governing bodies of mega sport organizations. However, specific training, capacity building, investment in all sections of governing bodies within an organization and its procedural matters require basic human rights. It would be most important that mainstreaming contributes to developing organizational probity and genuine commitment for integration of Ruggie Principle within economic aspects of governing bodies.

Whilst development of human rights and other units like anti-discrimination among FIFA & IOC to be welcomed all guiding principles. The established standards of human rights through guiding principles and other initiatives ensure security and remedial measures at the workplace in mega sport events and within the partners [52]. To understand and ensure the complete human rights impact assessment fulfilled the possible activities and due diligence of IOC along with FIFA organizing events, and also initiate mainstreaming to anticipatory. The primary aim is to complete available approaches and their compliance ensure better, reliable and comprehensive implementation of human rights aspects during the mega sport events.

## 6 Implication of research

The research on the promotion of human rights in mega-sporting events, as viewed through the lens of the Ruggie principle1, significantly influences practice and policy within the governance structures of the International Olympic Committee and FIFA. Specifically, the examination of the two governing bodies' practices and their application of human rights standards in major events like the Olympics and FIFA World Cup reveals crucial areas for enhancement and elements that require reconsideration or modification. As a result, the research emphasizes the importance of conducting an HRIA and incorporating it into the essence of sports organizations' processes.

Key findings show that due diligence and proactive management play a crucial role in reducing the negative consequences of human rights risks connected to mega events. The given source outlines an array of examples to demonstrate to what extent these governing bodies can succeed or fail, adhering to the Ruggie Principle's provisions and regulations while dealing with the issue and, thus, affecting the rights of the athletes, workers, and local communities involved. Similarly, the source analyzes the absence of systems throughout the bidding process and focuses on identifying approaches that could potentially improve the situation. Finally, the source emphasizes the importance of mainstreaming in the Ruggie Principle for business and human rights, which plays a crucial role in establishing a more systematic approach to addressing the issue of human rights.

Moreover, being interdisciplinary in nature, the research is beneficial for a broader understanding of the relationship between sports governance, human rights, and international law. The existing theoretical foundation serves as an impetus for the development of future research areas. The latter might include research focusing on the means to secure accountability, transparency, and ethical standards within mega-sports organizations. Such a study should also aim at proposing policy recommendations corresponding to international human rights standards and guaranteeing the effectiveness of the current and future regulatory systems for mega events. As a result, this research can serve as a guideline for the practices of both the IOC and FIFA, as well as stakeholders, policymakers, and researchers striving to protect human rights while organizing large sporting events. The study contributes to closing the gap between theory and practice by promoting ethical sports governance, safeguarding human rights, and laying the groundwork for a more inclusive and responsible approach to sports governance.

## 7 Conclusion

The provided article offers a profound comprehension of the connections between sports bodies with regard to the Ruggie Principles and mega sports events. The specified material emphasizes the fact that systemic processes are required to manage human rights and ensure due diligence and impact assessments, which have become vitally important for such organizations as FIFA and the IOC. Integrating human rights into these bodies' key processes is crucial and should be considered a priority. According to the article's key findings, there is a need for ongoing improvement in the specified structure of mega sports event organizations in terms of human rights. The provided research identifies ways to improve implementation and facilitate compliance with human rights standards in sports bodies.

This study offers valuable insights into potential SDG implementation strategies, such as incorporating due diligence and human rights impact assessments into the Olympic Games' operational framework. The study also provides a comprehensive overview of the challenges associated with achieving the goals, featuring sociological, human rights, and economic perspectives. Thus, the research can consider it a response to the Ruggie Report's requirement to conduct research that enhances our understanding of the practical application of the Ruggie Principles in the context of organizing mega sports events. Still, the readers have to keep investigating the issue of complexity and the interaction between human rights and sports, which is why this information may be vital. This research provides critical information about the opportunities for the sport's governing bodies to improve human rights. The stated requirements require participation from sport and event organizations, and those who are willing to oversee them adhere to legal norms, including human rights advocates, non-governmental organizations, sponsors, and investors. Such bodies as the IOC and FIFA should also continue with their net assessment processes, implementing changes to conform to the Ruggie Principles.

Further studies on this topic should be based on the present research. In such a way, they might include an analysis of new case studies related to mega-sports events that support the evidence presented in the findings section. More case studies might be useful to prove the advantages of the Ruggie Principles for organizations conducting mega sports events around the world. The discussion might also include studies of the long-term effects of using the Ruggie Principles on countries hosting such events. It is also possible to compare different international sports federations and their approaches to human rights protection. These studies might also focus on the use of information technologies to enhance transparency. Finally, further studies might investigate the potential of the collaboration between sports organizations and civil society in this area.

## Funding statement

The research received no external funding.

## Ethical approval

This article does not contain any studies with human participants performed by any of authors.

## Informed consent

This article does not contain any studies with human participants performed by any of authors.

## Data availability statement

Some or all data that support the findings of this study are available from the corresponding author upon reasonable request.

## CRediT authorship contribution statement

**Shuang Lin:** Methodology, Investigation, Funding acquisition. **Asif khan:** Writing – review & editing, Writing – original draft. **Yu Song:** Supervision, Resources, Methodology.

## Declaration of competing interest

The authors declare that they have no known competing financial interests or personal relationships that could have appeared to influence the work reported in this paper.

## Footnotes

1 Source: own extracted.

2 own extracted.

3 own extracted.

4 own extracted.
